# Supplementary material for: Expression site attenuation mechanistically links antigenic variation and development in Trypanosoma brucei
Source: eLife. 2014 May 20;3:e02324. doi: 10.7554/eLife.02324 (PMC4027811; doi:10.7554/eLife.02324)
Supplement: Supplementary file 1. — Cell lines and targeting constructs. DOI: http://dx.doi.org/10.7554/eLife.02324.016 [file elife02324s001.docx]

| Cell Line | Targeting Construct | Integration Site | 5’UTR | Insert | 3’UTR | Selectable Marker |
| --- | --- | --- | --- | --- | --- | --- |
| 221^ES^.121^tet^ | pRS.121 | rDNA spacer | *EP* | *VSG121* | *VSG121* | *BLE* |
| ∆dot1b.121^tet^ | pRS(pur).121 | rDNA spacer | *EP* | *VSG121* | *VSG121* | *PUR* |
| Luc^ES^ | pkD.Luc | 221ES (telo) | *ACTIN* | *LUC* | *ACTIN* | *NEO* |
| GFP^EStel^.221^ES^.121^tet^ | pkD.GFP | 221ES (telo) | *VSG221* | *GFP* | *VSG221* | *NEO* |
| GFP^ESpro^.221^ES^.121^tet^ | p3845 | 221ES(prom) | *VSG221* | *GFP* | *VSG221* | *BLAS* |
| Luc^rPro^.221^ES^.121^tet^ | pRib.Luc | rDNA | *PARP* | *LUC* | *PARP* | *PUR* |
| Luc^tub^.221^ES^.121^tet^ | pTub.Luc | *β-TUBULIN* | *ALDOLASE* | *LUC* | *ALDOLASE* | *PUR* |
| GFP:PAD_utr_ | p4231 | *α-β-TUBULIN* | *TUBULIN* | *MS2::GFP::NLS* | *PAD1* | *BLAS* |
| ESAG1^RNAi^ | p2T7.ESAG1 | 177bp-repeats | *-* | *ESAG1* | *-* | *BLE* |
| ESAG2^RNAi^ | p2T7.ESAG2 | 177bp-repeats | *-* | *ESAG2* | *-* | *BLE* |
| ESAG3^RNAi^ | p2T7.ESAG3 | 177bp-repeats | *-* | *ESAG3* | *-* | *BLE* |
| ESAG6/7^RNAi^ | p2T7.ESAG6/7 | 177bp-repeats | *-* | *ESAG6/7* | *-* | *BLE* |
| ESAG8^RNAi^ | p2T7.ESAG8 | 177bp-repeats | *-* | *ESAG8* | *-* | *BLE* |
| ESAG12^RNAi^ | p2T7.ESAG12 | 177bp-repeats | *-* | *ESAG12* | *-* | *BLE* |
| Tubulin^RNAi^ | p2T7.Tubulin | 177bp-repeats | *-* | *α-TUBULIN 5'UTR* | *-* | *BLE* |

**Table S1. Cell lines and targeting constructs.**

221ES (prom): promoter region of the *VSG221* expression site; 221ES (telo): downstream of *ΨVSG221* in the *VSG221* expression site; *LUC*: luciferase; *BLE*: phleomycin; *NEO*: neomycin; *PUR*: puromycin; *BLAS*: blasticidin.
